# Influence of breed on physicochemical properties of meat from capons

**DOI:** 10.1016/j.psj.2025.105919

**Published:** 2025-10-01

**Authors:** J. Calik, J. Obrzut

**Affiliations:** National Research Institute of Animal Production, Department of Poultry Breeding, 32-083 Balicek. Krakowa, Poland

**Keywords:** Capon, Native breeds, Meat quality

## Abstract

The aim of this study was to evaluate the meat quality of capons of four breeds included in the genetic resources conservation program in Poland. The experimental material consisted of 160 birds of the breeds Rhode Island Red (R-11), Rhode Island White (A-33), Sussex (S-66), and Yellow-legged Partridge (Ż-33), which were randomly assigned to four groups of 40 birds in each. These breeds with varied phenotype and productivity are characterized by good health and resistance to unfavorable climatic conditions, hence they are recommended for backyard farming. Extensive production mostly uses hens for laying production, and cockerels are mostly hatchery waste. All birds were fed *ad libitum* with identical feed mixtures and kept on bedding under optimal environmental conditions. The castration procedure was carried out at 8 weeks of age under local anesthesia by a veterinarian. After the end of fattening at 24 weeks of age, 10 birds with body weights close to the group average were selected for slaughter from each group. After slaughtering, the birds’ dressing percentage, physicochemical parameters of breast and leg muscles were determined, and sensory evaluation was carried out. The results provide valuable information on the formation of performance parameters and meat quality of capons of different breeds. Rhode Island Red (R-11) capons compared to Rhode Island White (A-33), Sussex (S-66), Yellow-legged Partridge (Ż-33) breeds were characterized by significantly highest body weight, dressing percentage and carcass muscularity. Both the breast and leg muscles of Rhode Island Red (R-11) and Yellow-legged Partridge (Ż-33) capons, compared to the other breeds, were characterized by better physicochemical parameters and higher sensory score, which is evidence that the above breeds can be considered as the best for capon production.

## Introduction

The progressive intensification of poultry production, especially egg-laying production, is associated with an increase in the number of surplus cockerels, which are generally disposed of immediately after hatching, raising major ethical questions (Busse et al., 2019). In recent years, there has been clear public opposition to the practice of killing day-old chicks, hence various alternatives for their management are being sought. Murawska et al. (2005) proposed the management of laying-type cockerels for meat use. However, due to the high feed consumption per kg of gain and inferior slaughter value, it was concluded that the profitability of rearing cockerels compared to slaughter chickens was not justified. Similar findings were presented by Koening et al. (2012), Giersberg and Kemper (2018) and Gautron et al. (2021). Another solution to this problem may be to castrate/caponize cockerels and fatten them to obtain meat with superior taste (Amorim et al., 2016; Calik et al., 2017), especially since consumers are increasingly demanding a greater variety and high quality of poultry meat products, and capon meat meets these requirements. Many authors report that the castration procedure contributes to a change in the birds’ metabolism, which affects the growth, behavior and physicochemical parameters of the meat obtained (Sirii et al., 2009; Calik et al., 2015; Adamski et al., 2016; Zawacka et al., 2017; Kwiecień et al., 2018; Gesek et al., 2019; Murawska et al., 2019; Hossen et al., 2021; Hossain et al., 2022; Calik and Obrzut, 2023a; Kasperek et al., 2023; Sumon et al., 2024). It has also been observed that capons have an increased content of adipose, subcutaneous and especially intramuscular fat, which in turn has a beneficial effect on enhancing the flavor, texture and juiciness of the meat (Tor et al., 2002; Chen et al., 2006; Miguel et al., 2008; Volk et al., 2011; Simeon et al., 2012; Kwiecień et al., 2015; Amorim et al., 2016; Gesek et al., 2017; Calik et al., 2017, 2020).

Today, the castration procedure is performed in many countries in Europe, Asia and America, and capons are marketed as higher quality products and fetch higher prices than broiler chicken and organic chicken meat (Simeon et al., 2010; Franco et al., 2016). The authors emphasize that native or locally adapted chicken breeds are most often used for capon production, and the efficient use of domestic chicken resources is the best form of conservation. They are a valuable source of products, which is a niche production, but forms an important element of diversification for consumers, particularly since in recent years there has also been a growing interest in products from slaughter chickens produced based on native breeds reared under extensive conditions (Diaz et al., 2010; Calik, 2014; Kwiecień et al., 2015; Faraji-Arough et al., 2019; Kasperek et al., 2021). The National Research Institute of Animal Production (Poland) has a unique collection of breeds/strains of laying hens, most of which are included in the inventory of global genetic resources (World Watch List for Domestic Animal Diversity, 2000). Among them, the native hen breed Yellow-legged Partridge (Ż-33) and locally adapted breeds Rhode Island Red (R-11), Sussex (S-66) and Rhode Island White (A-33) hold a special place. These breeds with varied phenotype and productivity are characterized by good health and resistance to unfavorable climatic conditions, hence they are recommended for backyard farming (Calik, 2008; Puchała et al., 2014; Krawczyk and Calik, 2018; Calik and Obrzut, 2023b). Extensive production mostly uses hens for laying production, and cockerels are mostly hatchery waste. Evaluation of production parameters as well as meat quality of capons, with feeding based on domestic protein sources will indicate the breeds most predisposed to this type of production.

The aim of the study was to compare the physicochemical quality characteristics of capon meat of four breeds covered by a conservation program in Poland.

## Material and methods

### Birds, maintenance and diets

The experiment was carried out in compliance with the European Union law (Directive 2010/63/UE, approved in Poland by Legislative Decree 266/2015) of the European Parliament and of the Council on the protection of animals used for scientific purposes. All procedures used during the study were approved by the 1st Local Ethical Committee for Animal Experiments in Kraków, Poland (Consent No. 515/2021 dated 28 April 2021).

The experimental material consisted of Rhode Island Red (R-11), Rhode Island White (A-33), Sussex (S-66), and Yellow-legged Partridge (Ż-33) birds. Chicks on the first day of life, after being weighed and individually labeled, were randomly assigned to 4 groups of 40 birds (5 replicates of 8 chicks) in each breed. The vaccination program included vaccination against Marek’s disease, Newcastle disease, infectious bronchitis, Gumboro disease and coccidiosis. All breeds/strains were maintained under welfare-friendly and optimal environmental conditions. Birds were kept in a litter system, at a stocking rate of 5 birds/m^2^. The environment in which the birds were housed was enriched with additional elements, i.e. perches, extra bales of straw, a scratching space, a sand-bathing and claw-trimming area, and grit. Initially, the chicks were provided with a temperature under the artificial brooder of about 32°C, and then as the birds grew older it was gradually lowered so that after 35 days of age until the end of fattening it was 17–20°C. The relative humidity in the room was between 65 and 70 %. The light program was 20–24 h (40 lux) until day 14, followed by 12–16 h (5–10 lux). Throughout the rearing period, the birds were fed *ad libitum* and provided with constant unlimited access to water. Up to and including the 7th week of rearing, they were fed a loose mix containing 202 g of crude protein and 2820 kcal (11.80 MJ ME in 1 kg of feed); from the 8th to the 16th week a mixture containing 184 g of crude protein and 2880 kcal (12.05 MJ ME); and from the 17th to the 24th week a mixture containing 165 g of total protein and 2905 kcal (12.15 MJ ME). The feed mixtures were prepared using grain meal based on domestic sources excluding soybean meal. The component composition and results of chemical analysis of the feed mixtures, according to AOAC (2000) methodology, are shown in Table 1.

**Table 1:** Composition and nutrient content of the diets used in the experiment (kg/100 kg).

Component	Phase I day 1 to wk 7	Phase II wk 8 to 16	Phase III wk 17 to 24
Ground maize	43.50	50.00	49.50
Ground wheat	10.00	10.17	19.70
Ground beans	9.00	9.00	8.00
Ground yellow lupine	14.00	11.00	10.00
Post-extraction rapeseed meal	13.00	10.00	6.00
Feed yeast	5.00	5.00	3.00
Rapeseed oil	2.00	1.50	0.50
Feed chalk	1.10	1.10	1.05
Dicalcium phosphate	1.50	1.35	1.40
DL-Metionina	0.10	0.08	0.05
NaCl	0.30	0.30	0.30
Vitamin-mineral premix DKA-F (finisher) (0.5%), kg	0.50	0.50	0.50
Crude protein (g)	202	184	165
Metabolizable energy (MJ) (kcal)	11.80 2820	12.05 2880	12.15 2905
Lys (g)	9.80	8.83	7.35
Met (g)	3.95	3.58	2.95
Ca (g)	8.95	8.50	7.95
P available (g)	4.10	3.80	3.50

The castration procedure for all cockerels was carried out at 8 weeks of age by a qualified veterinarian under local anesthesia, with full surgical asepsis, according to Commission Regulation (EC) No. 543/2008. The rearing of capons lasted until 24 weeks of age. During the experiment, data on bird health and feed consumption per kg of weight gain were recorded (Table 2).

**Table 2:** Mortality and health cullings and use feed on 1 kgs of body weight.

Item Strains	Mortality and health cullings					Feed conversion ratio (FCR - kg/kg) x±SD
	Number of birds				%	
	0-8 wk	9-12 wk	13-20 wk	Total	x±SD	
R-11	-	-	-	0	0.00 ± 0.00	4.98^a^ ± 0.04
A-33	-	-	-	0	0.00 ± 0.00	5.49^b^ ± 0.05
S-66	-	-	-	0	0.00 ± 0.00	5.29^ab^ ± 0.03
Ż-33	2	-	-	2	5.00 ± 0.50	5.20^ab^ ± 0.04

Notes: SD, standard deviation; x, mean value; Strains: R-11, Rhode Island Red; A-33, Rhode Island White; S-66, Sussex; Ż-33, Yellowleg Partridge.

a, b - values in rows with different letters differ significantly (*P*≤0.05).

A, B - as above for *P*≤0.01.

### Collection of samples

At 24 weeks of age, 10 capons (from 5 replicates of 2 pieces) with body weights close to the average in the group were selected from each group for slaughter. The birds did not receive feed for about 12 h before slaughter, while they were provided with constant access to water. The killing act was carried out by a trained person, using the decapitation method. The slaughtering procedure was in accordance with EU Regulation No., 1099/2009 of 24 September 2009 on the protection of animals at the time of killing. The pH of the breast and leg muscles was then determined at 15 min after the birds were slaughtered, and standard post-slaughter processing was carried out, i.e. scalding, defeathering and evisceration of the birds. All bird carcasses were weighed, their color was determined (whole carcass with skin), and then cooled at 4 °C. After 24 h of chilling, whole carcasses were subjected to a simplified analysis according to the methodology of Ziołecki and Doruchowski (1989), determining dressing percentage with giblets, proportion of breast and leg muscles, bones, giblets (gizzard, liver, heart) and abdominal fat. Whole breast and leg muscles were dissected from each carcass and sampled to determine their pH, color, water holding capacity, drip loss, cooking loss and tenderness. Sensory evaluation of the breast and leg muscles included parameters such as aroma, juiciness, tenderness and flavor. Chemical analysis of the muscles was also carried out, determining the levels of water, total protein, crude fat, crude ash, collagen, cholesterol and fatty acids.

### Analytical methods

#### Physical analysis

The acidity of breast and leg muscles determined at pH 15 min and pH 24 h after slaughter was measured using a CyberScan 110 pH meter with a Hamilton glass electrode calibrated to pH 4.0 and 7.0 (Eutech Instruments Pte Ltd/Oakton Instruments). Carcass and capon muscle color was determined in the L*a*b* color space (CIE, 2007) using a Minolta CR 410 reflectance spectrophotometer (Konica Minolta Holdings, Inc., Japan; light source D65, observer 2°). The colorimeter was calibrated throughout the study using a standard white ceramic plate (reference number 20633065; *Y* = 86.40, *x* = 0.3180 and *y* = 0.3350). The carcass colour result is the mean of five whole bird measurements (1 measurement from the dorsal part, 2 measurements from the thoracic part, 2 measurements from the thigh part of the legs), while the muscle colour is the mean of 2 measurements of the breast muscle and 2 femoral muscle measurements taken on the inner surface immediately after bone separation. Water holding capacity (WHC) of breast and thigh muscles was determined using the Grau and Hamm method (1953) based on the amount of juice mechanically pressed from the sample onto a filter paper (Whatman Schleicher & Schuell Grade 1 Qualitative, Cat No 1001 917, Whatman International Ltd Maidstone, England), and the leakage area was estimated using a planimeter (Haff Digital Polar Planimeter No. 301, Germany). Drip and cooking loss of the meat was determined after storing the samples of breast and thigh muscles for 24 h at 4 °C. In order to do this, 80 g meat samples were collected from the thigh and the breast muscle which were then placed in tightly sealed containers and stored in a refrigerator. Cooking loss was determined as the loss in weight of breast and thigh muscles during cooking. 80 g samples were placed in plastic bags and cooked at 100°C for 14 min (breast muscles) and 16 min (thigh muscles) to an internal temperature of 78°C. After cooking, the samples were chilled at room temperature for 30 min and then in a refrigerator at 4°C for 45 min. The measurement of muscle tenderness was performed using the TA.XT plus texture analyzer (Stable Micro Systems Ltd, Godalming, Surrey, GU71YU, UK). In order to do this, samples 10 mm in diameter and 30 mm in height were cut out from the cooked breast and thigh muscles (78°C). The collected sample was cut with a Warner-Bratzler knife (2 mms^-1^) in three places, perpendicularly to the course of muscle fibers, while the final measurement result was provided as a mean value.

#### Chemical analysis

Samples of breast and thigh muscles were collected from 6 birds of each group in order to determine the chemical composition, i.e. the contents of total dry matter, total protein (Kjeldahl method) and crude fat (Soxhlet method) and collagen using the AOAC method (2000). Cholesterol content was determined using gas chromatography (Shimadzu GC-2010 Plus). Fatty acid content was determined by gas chromatography (VARIAN 3400 CX) using helium as a carrier gas and column Rtx 2330 (105 m). Injector temperature was 200°C and detector temperature 240°C. The samples were prepared by the method of Folch et al. (1957) using BF3/methanol methylation. Chemical analyses were performed at the Central Laboratory of the Institute.

#### Sensory analysis

The sensory evaluation of breast and thigh muscles was conducted according to the methodical assumptions of Baryłko-Pikielna and Matuszewska (2009). The sensory evaluation test was carried out on meat samples cooked without salt or spices to an internal temperature of 80°C. After cooking, the muscle samples were cut into 1 × 2 cm pieces, protected with aluminum foil, and marked with an identification number. Next, the samples were served for tasting to a sensory panel consisting of 10 people. The following parameters were taken into account in the evaluation: aroma, juiciness, tenderness and taste (*n* = 10). The team of people included experienced and specially trained employees of the Institute. A 5-point scale from 1 to 5 points (with an accuracy of 0.5 points) was used, with a rating of 5 being the best, and 1 being the worst.

#### Statistical analysis

The Shapiro-Wilk test was applied to assess the normality of the data distribution. The results were then statistically processed using one-way ANOVA in Statistica 13,1. Significant differences in means between experimental groups were determined by Duncan’s test. Effects were considered significant at probabilities of *p* ≤ 0.05 and *p* ≤ 0.01.

## Results

As can be seen from Table 2, during the fattening period of the capons, mortality and health-related culling ranged from 0.00 (R-11, A-33, S-66) to 5.00 % (Ż-33). The lowest feed intake per kg of weight gain was recorded in R-11 capons (4.98 kg), while the highest in A-33 capons (5.49 kg), at *P* ≤ 0.05.

The results of body weight and slaughter analysis of cockerels and capons are shown in Table 3. The average body weight at 24 weeks of age was significantly (*P* ≤ 0.01) the highest in R-11 capons (2424 g), with significantly (*P* ≤ 0.01) the highest dressing percentage with giblets (72.26 %). The lowest body weight and dressing percentage was recorded in A-33 capons, which was 219 g and 2.79 percentage points lower, respectively. R-11 capons, compared to A-33, S-66 and Ż-33, were characterized by significantly (*P* ≤ 0.05 and *P* ≤ 0.01) higher breast muscle (17.47 vs. 15.81 - 16.24 %), leg muscle (22.40 vs. 20.82 - 21.29 %) and bone proportion (6.23 vs. 5.61–6.08 %). This breed also had a higher content of giblets, especially liver, at *P* ≤ 0.05. Abdominal fat content accounted for 2.01 (Ż-33) to 2.34 % (S-66) of carcass weight, with significant statistical differences (*P* ≤ 0.05). There was a wide variation in carcass color (*P* ≤ 0.05 and *P* ≤ 0.01). The highest values of L* (lightness) were recorded in A-33 capons (L* = 72.19), and significantly (*P* ≤ 0.05) lower values were recorded in Ż-33 capons (L* = 69.95). With regard to redness (a*), the highest value was recorded in the A-33 and S-66 strains (a* = 4.20 - 4.45), while for yellowness (b*) in the R-11 and Ż-33 capons (b* = 13.99 - 15.31), at *P* ≤ 0.05 and *P* ≤ 0.01, respectively.

**Table 3:** Body weight and results of slaughter analysis.

Item		Strains x±SD			
		R-11	A-33	S-66	Ż-33
Live body weight (g)		2424^A^ ± 104.60	2205^Ba^ ± 118.92	2301^Bb^ ± 55.66	2255^B^ ± 57.32
Dressing percentage with giblets (%)		72.26^A^ ± 0.24	69.47^B^ ± 1.28	71.48^A^ ± 1.47	71.35^A^ ± 0.90
Content in carcass breast muscles (%)		17.47^A^ ± 0.43	15.81^B^ ± 0.26	16.20^B^ ± 1.13	16.24^B^ ± 1.22
Content in carcass leg muscles (%)		22.40^Aa^ ± 0.74	20.82^B^ ± 0.87	21.29^b^ ± 1.21	21.24^b^ ± 0.75
Leg bones (%)		6.23^Aa^ ± 0.38	5.78^bc^ ± 0.39	6.08^b^ ± 0.32b	5.61^Bc^ ± 0.27
Giblets (%)		5.40^a^ ± 0.14	5.08^b^ ± 0.26	5.23^ab^ ± 0.37	5.13^ab^ ± 0.32
Liver (%)		2.32^a^ ± 0.13	2.07^b^ ± 0.09	2.18^ab^ ± 0.21	2.12^ab^ ± 0.16
Gizzard (%)		2.54 ± 0.14	2.47 ± 0.24	2.51 ± 0.19	2.46 ± 0.14
Heart (%)		0.54 ± 0.01	0.53 ± 0.03	0.54 ± 0.04	0.52 ± 0.04
Abdominal fat (%)		2.09^b^ ± 0.28	2.06^b^ ± 0.65	2.34^a^ ± 0.26	2.01^b^ ± 0.46
Carcass color	L*	71.31^ab^ ± 1.22	72.19^a^ ± 1.67	71.51^ab^ ± 1.54	69.95^b^ ± 2.12
	a*	3.19^b^ ± 0.92	4.20^a^ ± 0.81	4.45^a^ ± 0.77	3.28^b^ ± 0.75
	b*	13.99^A^ ± 3.17	11.31^B^ ± 2.40	9.90^B^ ± 1.67	15.31^A^ ± 2.47

Notes: SD, standard deviation; x, mean value; Strains: R-11, Rhode Island Red; A-33, Rhode Island White; S-66, Sussex; Ż-33, Yellowleg Partridge.

a, b - values in rows with different letters differ significantly (*P* ≤ 0.05);.

A, B - as above for *P* ≤ 0.01.

Physical parameters of breast and leg muscles are presented in Table 4. The evaluated breeds/strains of capons did not differ significantly in terms of breast and leg muscle pH measured at 15 min. However, in the measurement made after 24 h (pH_24_) in both breast and leg muscles, the lowest value of this indicator was found in A-33 capons, and the differences in this regard compared to the R-11, S-66 and Ż-33 strains were confirmed statistically (*P* ≤ 0.05). In evaluating color, it was found that the breast muscles of R-11, A-33 and S-66 capons, unlike those of the Ż-33 strain, were significantly (*P* ≤ 0.05) lighter (L*), while those of A-33 and S-66 were characterized by significantly (*P* ≤ 0.01) higher redness (a*). In terms of yellowness (b*), among the breeds evaluated, capons R-11 and Ż-33 stood out, in contrast to those of the A-33 and S-66 strains (*P* ≤ 0.05). Similar correlations were noted for leg muscle color, which in capons A-33 and S-66, in contrast to Ż-33 and R-11, was significantly (*P* ≤ 0.05) lighter (L*) and more red (a*), while having the lowest yellowness (b*). There were favorable and significant (*P* ≤ 0.05) statistical differences between the R-11 and Ż-33 and A-33 strains for breast and leg muscles in terms of drip loss, cooking loss and water holding capacity. The best tenderness parameters were exhibited by the breast and leg muscles of Ż-33 (13.52 and 17.20 N) and R-11 capons (14.15 and 18.32 N) in contrast to A-33 capons (17.28 and 22.71 N), with significant statistical differences (*P* ≤ 0.05).

**Table 4:** Technological parameters of breast and leg muscles.

Item	Breast muscles x±SD				Leg muscles x±SD			
	Strains				Strains			
	R-11	A-33	S-66	Ż-33	R-11	A-33	S-66	Ż-33
pH_15_	6.48 ± 0.11	6.40 ± 0.10	6.42 ± 0.08	6.46 ± 0.05	6.55 ± 0.05	6.47 ± 0.05	6.58 ± 0.06	6.58 ± 0.06
pH_24_	6.03^a^ ± 0.12	5.67^b^ ± 0.10	5.99^a^ ± 0.05	6.01^a^ ± 0.09	6.08^a^ ± 0.06	5.71^b^ ± 0.06	6.07^a^ ± 0.03	6.10^a^ ± 0.10
Color muscles:								
L*	58.92^a^ ± 3.21	58.60^a^ ± 1.18	58.79^a^ ± 1.98	56.24^b^ ± 2.99	47.93^a^ ± 5.72	47.66^a^ ± 1.51	48.39^a^ ± 3.23	44.73^b^ ± 2.24
a*	11.27^A^ ± 1.25	13.49^B^ ± 0.73	13.89^B^ ± 0.72	11.39^A^ ± 1.09	15.19^a^ ± 4.06	18.91^b^ ± 1.38	19.03^b^ ± 0.47	14.46^a^ ± 1.09
b*	12.64^a^ ± 1.79	10.13^b^ ± 0.33	10.21^b^ ± 1.56	12.40^a^ ± 1.95	9.03^b^ ± 2.55	7.51^a^ ± 1.44	7.18^a^ ± 1.87	9.39^b^ ± 1.52
Drip loss after 24 h (%)	0.40^a^ ± 0.08	0.49^b^ ± 0.10	0.45^ab^ ± 0.08	0.39^a^ ± 0.09	0.19^a^ ± 0.03	0.27^b^ ± 0.06	0.23^ab^ ± 0.06	0.17^a^ ± 0.03
Thermal loss (%)	21.56^a^ ± 2.25	23.23^b^ ± 1.61	21.96^ab^ ± 2.31	21.35^a^ ± 2.42	27.65^a^ ± 3.84	30.74^b^ ± 2.76	29.83^ab^ ± 1.89	27.71^a^ ± 2.03
Water holding capacity (%)	12.02^a^ ± 1.01	14.36^b^ ± 1.51	13.38^ab^ ± 1.01	11.62^a^ ± 1.77	14.19^a^ ± 2.10	16.59^b^ ± 1.73	15.67^ab^ ± 1.63	13.92^a^ ± 0.92
Tenderness (N)	14.15^a^ ± 2.18	17.28^b^ ± 3.57	15.31^ab^ ± 2.46	13.52^a^ ± 2.10	18.32^a^ ± 2.95	22.71^b^ ± 3.61	20.04^ab^ ± 2.77	17.20^a^ ± 2.14

Notes: SD, standard deviation; x, mean value; Strains: R-11, Rhode Island Red; A-33, Rhode Island White; S-66, Sussex; Ż-33, Yellowleg Partridge. a, b - values in rows with different letters differ significantly (*P* ≤ 0.05); A, B - as above for *P* ≤ 0.01.

In sensory evaluation (Table 5), both breast and leg muscles of all evaluated capon breeds scored high in all evaluation categories, i.e., color, aroma, juiciness and tenderness. Evaluators gave particularly high marks to leg muscles from R-11 and Ż-33 capons.

**Table 5:** Results of sensory analysis of breast and leg muscles.

Item	Breast muscles x±SD				Leg muscles x±SD			
	Strains				Strains			
	R-11	A-33	S-66	Ż-33	R-11	A-33	S-66	Ż-33
Aroma (pts.)	4.65 ± 0.32	4.50 ± 0.38	4.45 ± 0.41	4.70 ± 0.33	4.70 ± 0.25	4.60 ± 0.27	4.55 ± 0.41	4.75 ± 0.25
Juiciness (pts.)	4.70 ± 0.33	4.65 ± 0.39	4.60 ± 0.44	4.75 ± 0.33	4.80 ± 0.25	4.60 ± 0.40	4.65 ± 0.31	4.85 ± 0.23
Tenderness (pts.)	4.65 ± 0.39	4.50 ± 0.38	4.40 ± 0.39	4.70 ± 0.38	4.80 ± 0.24	4.65 ± 0.48	4.65 ± 0.47	4.85 ± 0.25
Flavor (pts.)	4.75 ± 0.26	4.40 ± 0.37	4.45 ± 0.39	4.65 ± 0.35	4.85 ± 0.25	4.65 ± 0.31	4.75 ± 0.21	4.85 ± 0.25

Notes: SD, standard deviation; x, mean value; Strains: R-11, Rhode Island Red; A-33, Rhode Island White; S-66, Sussex; Ż-33, Yellowleg Partridge.

The differences in terms of the content of basic chemical constituents in the muscles are shown in Table 6. In the breast muscles of Ż-33 capons compared to the other strains, there was a significantly (*P* ≤ 0.05 and *P* ≤ 0.01) higher content of ash and a tendency toward higher crude protein content. Also, Ż-33 compared to S-66 capons in both breast and leg muscles had a significantly (*P* ≤ 0.05) lower fat and cholesterol content, and a lower collagen content compared to the A-33 breed. A significantly higher content of saturated fatty acids (SFA) and a lower content of unsaturated fatty acids (UFA) were recorded in the breast muscles of S-66 capons, and compared to A-33 capons these differences were statistically confirmed (*P* ≤ 0.05). In leg muscles, the differences were significant at the *P* ≤ 0.01 level between capons S-66 and R-11, A-33 and Ż-33. The ratio of n-6 and n-3 PUFAs in breast muscles ranged from 10.40 (R-11) to 13.34 (S-66), and in leg muscles from 15.22 (A-33) to 17.61 (S-66). For the other traits, the differences between the strains were statistically insignificant.

**Table 6:** Results of chemical analysis of the breast and leg muscles.

Item	breast muscles x±SD				leg muscles x±SD			
	Strains				Strains			
	R-11	A-33	S-66	Ż-33	R-11	A-33	S-66	Ż-33
Dry matter (%)	25.52 ± 0.36	25.43 ± 0.82	25.73 ± 0.56	25.56 ± 0.48	26.01 ± 0.56	25.52 ± 1.01	24.75 ± 0.76	25.42 ± 0.84
Crude ash (%)	1.10^a^ ± 0.02	1.09^a^ ± 0.01	1.08^A^ ± 0.02	1.12^Bb^ ± 0.02	1.02 ± 0.02	1.01 ± 0.01	1.02 ± 0.01	1.07 ± 0.10
Crude protein (%)	24.22 ± 0.37	24.39 ± 0.52	24.24 ± 0.48	24.68 ± 0.39	20.89 ± 0.38	20.34 ± 0.57	20.42 ± 0.53	20.93 ± 0.25
Crude fat (%)	0.94^ab^ ± 0.14	1.03^ab^ ± 0.46	1.23^a^ ± 0.16	0.90^b^ ± 0.40	4.29^ab^ ± 0.81	4.45^ab^ ± 0.71	4.77^a^ ± 0.59	3.78^b^ ± 0.30
Cholesterol (mg/100 g)	0.54^ab^ ± 0.06	0.55^ab^ ± 0.06	0.59^a^ ± 0.05	0.50^b^ ± 0.04	0.81^ab^ ± 0.09	0.82^ab^ ± 0.06	0.87^a^ ± 0.09	0.79^b^ ± 0.07
Collagen [g/100 g]	0.48^ab^ ± 0.09	0.51^a^ ± 0.10	0.47^ab^ ± 0.05	0.40^b^ ± 0.03	0.91^ab^ ± 0.12	1.09^a^ ± 0.14	1.04^ab^ ± 0.11	0.84^b^ ± 0.10
Fatty acid (g/100 g)								
C _14:0_	0.52^ab^ ± 0.04	0.56^a^ ± 0.07	0.52^ab^ ± 0.04	0.45^b^ ± 0.07	0.69^a^ ± 0.04	0.75^A^ ± 0.04	0.73^A^ ± 0.04	0.63^Bb^ ± 0.05
C _16:0_	23.43 ± 0.15	22.92 ± 1.38	24.49 ± 0.80	24.13 ± 1.98	22.51^a^ ± 0.21	22.33^a^ ± 1.36	24.30^b^ ± 1.09	23.21^ab^ ± 1.56
C _16:1_	2.22 ± 0.53	2.69 ± 1.42	2.99 ± 0.71	2.99 ± 1.84	4.07 ± 0.75	4.59 ± 1.68	5.06 ± 0.92	5.82 ± 2.64
C _18:0_	11.99 ± 1.06	11.42 ± 1.62	11.88 ± 0.71	11.36 ± 1.33	10.22 ± 0.72	9.74 ± 1.32	10.70 ± 0.60	9.73 ± 1.93
C _18:1_	30.45 ± 1.92	30.49 ± 2.27	29.21 ± 0.40	28.28 ± 4.04	36.11 ± 2.31	35.14 ± 2.01	33.96 ± 1.17	34.67 ± 2.46
C _18:2n-6_	16.48^a^ ± 1.29	19.14^b^ ± 2.18	16.20^a^ ± 2.07	16.03^a^ ± 2.10	21.01 ± 1.53	22.40 ± 2.87	19.93 ± 1.62	19.68 ± 2.60
C _18:3n-3_	0.64^a^ ± 0.06	0.83^Bb^ ± 0.19	0.61^a^ ± 0.14	0.43^A^ ± 0.11	1.04^ab^ ± 0.11	1.22^a^ ± 0.22	0.92^b^ ± 0.12	0.81^b^ ± 0.19
C_22_	0.13 ± 0.02	0.12 ± 0.04	0.15 ± 0.02	0.15 ± 0.04	0.05 ± 0.001	0.05 ± 0.001	0.05 ± 0.01	0.08 ± 0.08
C _20:4n-6_	11.83^ab^ ± 1.26	9.84^a^ ± 2.78	12.13^ab^ ± 2.02	14.07^b^ ± 4.45	3.63 ± 1.13	3.14 ± 1.40	3.73 ± 1.34	4.60 ± 1.75
C _22:6n-3_(DHA)	2.05 ± 0.42	1.67 ± 0.48	1.50 ± 0.57	1.82 ± 0.63	0.45 ± 0.18	0.41 ± 0.20	0.38 ± 0.17	0.56 ± 0.29
∑ SFA	36.23^ab^ ± 1.01	35.25^a^ ± 1.60	37.23^b^ ± 0.43	36.26^ab^ ± 1.41	33.65^A^ ± 0.62	33.04^A^ ± 1.76	35.95^B^ ± 0.68	33.81^A^ ± 0.84
∑ UFA	63.77^ab^ ± 1.01	64.75^a^ ± 1.60	62.76^b^ ± 0.44	63.74^ab^ ± 1.41	66.35^A^ ± 0.62	66.95^A^ ± 1.76	64.05^B^ ± 0.68	66.18^A^ ± 0.84
∑ MUFA	32.67 ± 2.21	33.19 ± 3.65	32.20 ± 0.70	31.28 ± 5.74	40.18 ± 2.69	39.73 ± 3.60	39.01 ± 1.73	40.49 ± 4.93
∑ PUFA	31.08 ± 1.59	31.56 ± 3.80	30.55 ± 0.95	32.45 ± 6.32	26.16 ± 2.45	27.23 ± 3.80	25.03 ± 2.32	25.69 ± 4.41
∑ PUFAn-6	28.32 ± 1.38	28.99 ± 3.36	28.33 ± 0.69	30.10 ± 5.85	24.64 ± 2.22	25.54 ± 3.52	23.67 ± 2.18	24.27 ± 4.01
∑ PUFAn-3	2.76 ± 0.37	2.57 ± 0.44	2.21 ± 0.51	2.35 ± 0.63	1.52 ± 0.25	1.69 ± 0.29	1.36 ± 0.20	1.41 ± 0.44
PUFAn-6/n-3	10.40 ± 1.33	11.38 ± 0.78	13.34 ± 2.93	13.25 ± 2.40	16.37 ± 1.75	15.22 ± 0.71	17.61 ± 2.06	17.21 ± 3.14

Notes: SD, standard deviation; x, mean value; Strains: R-11, Rhode Island Red; A-33, Rhode Island White; S-66, Sussex; Ż-33, Yellowleg Partridge. a, b - values in rows with different letters differ significantly (*P* ≤ 0.05); A, B - as above for *P* ≤ 0.01.

## Discussion

In the presented study, the prospects of capon meat production based on native breeds of hens were assessed. For this purpose, the health of the birds, feed intake per kg of weight gain and physicochemical parameters of meat were evaluated. The survival rate of the evaluated birds during the rearing period was at a good level. Mortality was observed only in Ż-33 capons and concerned the first days after the castration procedure was performed. Simeon et al. (2010) also showed low mortality rates, which were around 3.0 %. According to Rikimaru et al. (2009), caponizing losses are dependent on the weight and age of the birds at which the castration procedure is performed, and can increase up to 20 %. In our study, the procedure was performed at 8 weeks of age, and this timing was based on previous observations (Calik et al., 2015, 2017). Also, Shao et al. (2009) showed that caponization of cockerels in the first weeks of rearing (around week 6) is more beneficial due to their higher survival rate, better weight gain and carcass quality traits.

According to Commission Regulation (EC) No. 543/2008 of 16 June 2008, capons must be fattened for at least 77 days after castration and slaughtered at a minimum of 140 days. At the same time, the cited regulation does not specify the maximum permissible limit of the birds’ rearing time. In the presented study, capons were kept until the 24th week. Our own observations indicate that native breeds of hens reach sexual maturity later, around 23 weeks of age, and due to the relatively low weight gain as well as the greater fatness of the birds (abdominal and visceral fat) it is not economically justified to keep them longer. Also Duran (2004), evaluating the growth of cockerels and capons between 8 and 30 weeks of rearing, concluded that due to statistically significant differences in body weight, the most optimal slaughter date is the 25th week of rearing. Chen et al. (2007) and Shao et al. (2009) suggested terminating rearing between 22 and 26 weeks of age depending on the origin of the capons.

In the production of capons, nutrition plays a very important role. It is recommended that it be divided into 2 - 3 periods, and 70 % of their diet should be cereals, supplemented with roughage. In poultry nutrition, one of the most popular protein raw materials used in the production of compound feeds is extracted soybean meal, which is generally imported to Poland from South American countries and comes mainly from genetically modified (GM) soybean seeds. Concerns among some consumers about the use of feed from GM crops are forcing scientists to find alternatives to this raw material. In our previous studies conducted on Green-legged Partridge (Calik et al., 2015) and Rhode Island Red breeds (Calik et al., 2017), grain mixtures using extracted soybean meal were used. In order to meet the demands of consumers who are increasingly paying attention to the way birds are fed, only feed mixtures based on local protein sources were used in the presented studies. The results obtained indicate that the genotype of the bird has a strong influence on body weight. Among the breeds evaluated, R-11 capons stood out in particular, having the highest body weight and the best feed conversion per kg of gain during the 24-week fattening period. S-66, Ż-33 and especially A-33 capons had significantly lower body weights, as well as higher feed consumption, which, however, was only statistically confirmed between R-11 and A-33. The feed conversion ratio (FCR) is a measure of feed conversion efficiency, and its lower values in R-11 capons indicate more efficient feed utilization, which translates into lower production costs. Also, Chen et al. (2006) and Adamski et al. (2016) indicate that both bird origin and caponization can significantly affect utilization efficiency as expressed by feed consumption and conversion per kg of gain, and this variation was directly related to the weight of the birds in rearing. Faraji-Arough et al. (2019) indicate that the differences in body weights of native chickens of different breeds are much greater than those of commercial hybrid broiler chickens, but the quality of their meat is quite different, which can be of great importance to consumers. Hence, body weight should be one of the important criteria to consider when selecting breeds for caponization, as good growth rate has a direct impact on the profitability of production. In addition, as Rahman et al. (2004) and Sirri et al. (2009) point out, changes in appearance and behavior are observed in castrated birds as a result of testosterone deficiency. Castrated cockerels are less aggressive, so they can use the saved energy to convert feed more efficiently to increase body weight and deposit fat, including particularly valuable intramuscular fat (Franco et al., 2016; Kwiecień et al., 2018; Sumon et al., 2024).

In our study, R-11 capons, compared to the evaluated breeds A-33, S-66 and Ż-33, were also characterized by higher performance and carcass muscularity including higher breast and leg muscle content. This group also had a significantly higher content of giblets, especially liver, and the proportion of leg bones, which is consistent with the results of Rahman et al. (2004), Mahmud et al. (2013), Kwiecień et al. (2018) and Sumon et al. (2024). In the studies presented here, a lower proportion of breast muscles and a higher proportion of leg muscles was observed. As Wattanachant et al. (2004) point out, in broiler chickens these relationships are reversed as a result of the selection of these birds to gain more valuable breast muscle mass. Rikimaru et al. (2009) and Adamski et al. (2016) showed significant differences in carcass weight and dressing percentage depending on the origin of the birds. On the other hand, Jaturasitha et al. (2008) indicate that the origin of the birds has a significant effect on the proportion of breast and leg muscles in capon carcasses. In our study, the evaluated groups of capons differed in terms of abdominal fat, with the highest proportion in Sussex capons (S-66). Hossen et al. (2021) found that capons are characterized not only by a higher content of abdominal fat, but also subcutaneous and intramuscular fat, and that castration of cockerels lowers testosterone concentration and causes an increase in lipogenesis capability and lipid accumulation in the body, which in turn may affect the evaluated carcass color.

Color is the most significant and easily grasped quality characteristic of meat quality. It is an important trait that the consumer is guided by when making a purchase, as well as one of the most important indicators that determine the freshness and technological suitability of meat as a raw material (Sokołowicz et al., 2016). The evaluated groups of capons differed in terms of carcass and meat color. The most desirable color values were recorded for R-11 and Ż-33 capons, which, unlike A-33 and S-66 capons, were less red (a*) and more yellow (b*). Augustyńska-Prejsnar and Sokołowicz (2014) indicate that the content of heme pigments depends on bird genotype, age, sex, nutrition, type and activity of muscles, and that the color depends mainly on the content of myoglobin and intramuscular fat. Also cited authors state that an important factor on which the direction of physicochemical transformations depends is the pH of meat, which is an indicator of the variation in quality and technological suitability of the raw material. A great influence on the changes in the pH of muscle tissue is exerted by genetic, environmental factors and pre- and post-slaughter handling. Meat pH is closely related to color, water holding capacity and tenderness (Berri et al., 2005). Processing quality, on the other hand, is determined by drip loss, cooking loss and forced drip loss. In our study, we showed that muscle pH depends on the breed of capons. Breast and leg muscles of R-11 and Ż-33 capons, compared to A-33, were characterized by a significantly higher pH value, a more favorable water holding capacity, while at the same time positively lower drip and cooking losses. Połtowicz and Doktor (2012) emphasize that the water holding capacity of meat is one of the most important aspects from a technological as well as economic point of view. The cited authors point out that this characteristic has a significant impact on the juiciness and tenderness of meat, as well as on changes in the water content of meat during transport, refrigerated storage, and also during thermal processing. Cooking losses are undesirable due to loss of soluble components and reduction of meat juiciness, and the amount of losses during meat heating depends on the temperature and processing time, but also on the initial water and fat content of the meat (Choi et al., 2016). In our own research, it was shown that the tenderness of breast and leg muscles also depends on the breed of capons. In instrumental evaluation, the breast and leg muscles of R-11 and Ż-33 capons were more tender than those of A-33 capons. Wojtysiak et al. (2019) indicate that meat tenderness depends on the content, composition and structure of intramuscular connective tissue, but also on the degree of post-slaughter degradation of myofibrillar and cytoskeletal muscle fiber proteins. The authors emphasize that changes in the rate of protein degradation after death affect not only the structure of muscle fibers, but also the physicochemical parameters of meat. Sinanoglou et al. (2011), Volk et al. (2011) and Calik et al. (2017) show that the proportions of adipose tissue are significantly higher and the accumulation of subcutaneous and intramuscular fat more intense in capons than in uncastrated cockerels. Hence, the muscle tissue is characterized by better sensory qualities, i.e. juiciness, delicacy, tenderness and flavor, and thus the meat is more attractive. In the experiment conducted, despite the lack of statistical differences between the average scores for individual sensory characteristics, the muscles of R-11 and Ż-33 capons in particular, and especially leg muscles, were characterized by good quality. Against the background of previous studies (Calik et al., 2015, 2017), both breast and leg muscles scored high on all evaluated characteristics, i.e. aroma, juiciness, tenderness and flavor. As indicated by U-chupaj et al. (2017), the sensory characteristics of meat mainly depend on the age of the slaughtered birds, environmental factors and the method of thermal treatment, which is the main determinant affecting the development of a specific flavor and aroma profile of meat. The fat contained in the muscles is of great importance, as it reduces the drying of muscle tissue during heat treatment and promotes the feeling of juiciness. The cited authors emphasize that tenderness is expressed through the subjective sensation of hardness, resilience, or springiness of meat. Sokołowicz et al. (2016) report that meat from birds of native breeds is characterized by a more intense aroma and better flavor, and that the concentration of flavor precursors increases with the age of the birds. Also, as Augustyńska-Prejsnar and Sokołowicz (2014) point out, leg muscles are characterized by a stronger aroma than less active breast muscles, which was also observed in our study. Krawczyk et al. (2018) and Obrzut et al. (2018) demonstrated that meat from poulards, was more tender and scored better in sensory evaluation.

The nutritional value of meat is determined, among other things, by its protein and fat content. In the breast muscles of all the capon breeds studied, the level of total protein was above 24 % and was higher by 3 - 4 % compared to young chickens for slaughter (Wattanachant et al., 2004), but similar to the native capons Amarela Portuguesa and Pedręs Portuguesa (Amorim et al., 2016) and Green-legged Partridge and Polbar (Kwiecień et al., 2018). In the breast muscles of Ż-33 capons, a significantly higher ash content was recorded compared to the other strains, which is in line with the findings of Siria et al. (2009), Simeon et al. (2012) and Sumon et al. (2024). This group of capons also had significantly lower fat and cholesterol content compared to S-66 capons, and lower collagen content compared to A-33 capons. Similarly, correlations with respect to fat and collagen content were noted in leg muscles. The results of the studies by Calik et al. (2015), Amorim et al. (2016), Kwiecień et al. (2018) and Franco et al. (2016) show significantly higher protein and fat content in the muscles of sterilized birds. Against the background of the study by Wattanachant et al. (2004), the lipid content of breast and drumstick meat of capons was higher than that of broilers, and this fact can be attributed to the effects of sterilization on the cockerels’ metabolism. Sirii et al. (2009) showed higher cholesterol content in both breast and leg muscles in capons. As Janicki and Buzała (2013) point out, collagen has a significant effect on meat tenderness. The cited authors report that a lower collagen content is observed in the meat of late-maturing and castrated animals. In addition, the breast muscles of capons contain about 50 % less collagen compared to the thigh muscles (Amorim et al., 2016), which was also observed in our study. According to Diaz et al. (2010), the chemical composition of the meat of castrated cockerels does not depend on the breed, and only some parameters change to varying degrees with the age of the capons. The fatty acid content of the birds’ muscles is influenced by genotype, nutrition and the age of the birds, and the difference in fatty acid composition between breeds may be related to the different fat content of the muscles (Tor et al., 2005; Miguel et al., 2008). In our study, both the breast and leg muscles of sterilized S-66 birds showed a higher content of fat, saturated fatty acids (SFA) and a lower content of unsaturated fatty acids (UFA). Also, Kwiecień et al. (2018) showed a higher content of SFA in breast muscles in Polbar (Pb) capons. Sirri et al. (2009) reported a higher content of SFA in the breast muscles of capons without affecting the content of MUFA, PUFA n-3 and n-6 fatty acids. Our own research indicates in the breast muscles of R-11 capons a favorable trend from the point of view of human dietetics toward a higher content of n-3 PUFA and a lower ratio of PUFA n-6/n-3 fatty acids.

## Conclusion

Based on the results obtained, it was concluded that:

• The highest body weight, slaughter efficiency and musculature of the carcass were distinguished by the Rhode Island Red (R-11) capons.
• Among the breeds evaluated, both the breast and leg muscles of Rhode Island Red (R-11) and Yellow-legged Partridge (Ż-33) capons were characterized by better physical parameters of traits, i.e., carcass and muscle color, water holding capacity, drip loss, cooking loss and tenderness, and scored higher in sensory evaluation.
• In the pectoral muscles of the Yellow-legged Partridge (Ż-33) capons, a higher ash content was noted, and a lower fat and collagen content. On the other hand, Sussex (S-66) capon muscles had the highest content of saturated SFA acids and the lowest content of UFA acids.
• Among the assessed breeds, the best material for capon production are the Rhode Island Red (R-11) and Yellow-legged Partridge (Ż-33) breeds.

## CRediT authorship contribution statement

**J. Calik:** Writing – original draft, Validation, Methodology, Formal analysis, Data curation, Conceptualization. **J. Obrzut:** Methodology, Investigation, Data curation.

## Disclosures

The authors declare that they have no known competing financial interests or personal relationships that could have appeared to influence the work reported in this paper.
